# Severe Acute Pulmonary Toxicity Associated with Brentuximab in a Patient with Refractory Hodgkin's Lymphoma

**DOI:** 10.1155/2016/2359437

**Published:** 2016-04-17

**Authors:** Yasmin Sabet, Saul Ramirez, Elizabeth Rosell Cespedes, Marimer Rensoli Velasquez, Mateo Porres-Muñoz, Sumit Gaur, Juan B. Figueroa-Casas, Mateo Porres-Aguilar

**Affiliations:** ^1^Division of General Internal Medicine, Texas Tech University Health Sciences Center, El Paso, TX 79905, USA; ^2^Division of Medical Education, Paul L. Foster School of Medicine and Texas Tech University HSC, El Paso, TX 79905, USA; ^3^School of Medicine, Universidad Autonoma de Tamaulipas (UAT), Tampico, Mexico; ^4^Division of Hematology and Oncology, Texas Tech University Health Sciences Center, El Paso, TX 79905, USA; ^5^Division of Pulmonary and Critical Care Medicine, Texas Tech University Health Sciences Center, El Paso, TX 79905, USA

## Abstract

Acute pulmonary toxicity associated with brentuximab appears to be a rare but serious adverse effect that can be potentially fatal. We report the case of a twenty-nine-year-old female with Hodgkin's lymphoma who was treated with brentuximab and later presented with severe acute pulmonary toxicity; she improved after the discontinuation of brentuximab and administration of antibiotics and glucocorticoid therapy. Currently there is very little data in the literature in regard to the clinical manifestations and characteristics of patients taking brentuximab and the potential development of acute severe pulmonary toxicity, as well as the appropriate therapeutic approach, making this particular case of successful treatment and resolution unique.

## 1. Background

Brentuximab vedotin is a CD30-directed antibody-drug conjugate initially approved for the treatment of relapsed-refractory Hodgkin's lymphoma. In August 2015, on the basis of favorable progression free survival results noted in the phase III AETHERA trial, it was approved for consolidation therapy after autologous transplant in patients deemed to be of high risk of relapse [1]. As with many chemotherapeutic drugs, brentuximab may be associated with significant adverse effects. One of the rare but potentially life-threatening side effects is severe pulmonary toxicity. In the AETHERA trial pulmonary toxicity was reported in 5% of patients in the brentuximab arm [2].

## 2. Case Presentation

A twenty-nine-year-old female presented with a one-week history of progressive dyspnea, nonproductive cough, and chills. Initially her dyspnea was only with exertion but then progressed to dyspnea at rest. Her past medical history was significant for stage IV B Hodgkin's lymphoma diagnosed two years prior to clinical presentation. She was initially treated with six cycles of doxorubicin, bleomycin, vinblastine, and dacarbazine (ABVD) chemotherapy achieving complete radiological remission by PET (positron emission tomography) criteria.

However she relapsed after 4 months of completing ABVD and did not respond to salvage ifosfamide, carboplatin, and etoposide (ICE). She then received second line salvage therapy with dexamethasone, high dose cytarabine, and cisplatin (DHAP). Patient had a PET scan after DHAP chemotherapy. It showed complete radiological response with Deauville score of 2. She then underwent high dose chemotherapy with carmustine, etoposide, cytarabine, and melphalan (BEAM) followed by an autologous hematopoietic stem cell rescue. The patient was then started on consolidation therapy with brentuximab at a dose of 1.8 milligrams/kilogram on a three-week schedule while she was in complete remission and after her blood counts recovered. One week prior to her clinical presentation, the patient had received her third infusion of brentuximab after which she developed a nonproductive cough and diffuse pruritic rash that resolved with antihistamine administration.

**Figure 1:**
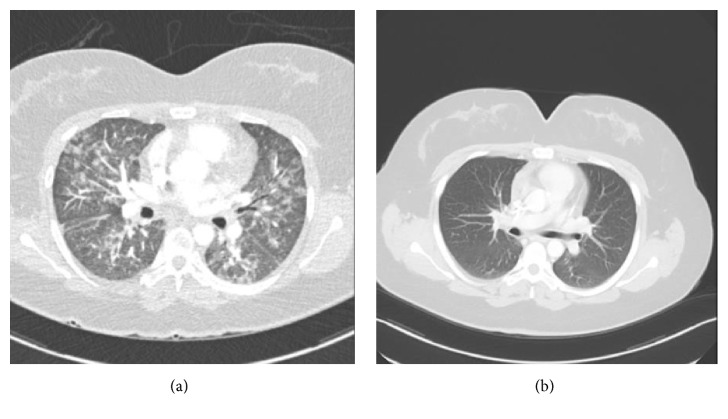
(a) Computed tomography (CT) of the chest showing patchy, nodular ground glass opacities along a bronchovascular distribution throughout both lungs. (b) CT of the chest showing resolution of previously seen opacities after discontinuation.

On the day of admission to our institution, remarkable physical findings included fever with core temperature of 38.4°C, heart rate of 105 beats per minute, and respiratory rate of 24 breaths per minute. Physical examination revealed a young female in moderate acute respiratory distress with shallow breathing and poor inspiratory effort. Lungs auscultation was significant for fine inspiratory crackles bilaterally and the rest of the physical examination was unremarkable. Laboratory workup was remarkable for a white blood cell count of 11,000 with a normal differential; absolute lymphocyte count was 1.63 × 103 UL. Her arterial blood gas values while on two liters of oxygen via nasal cannula were pH 7.4; pC02: 31.9; p02: 66.3 mmHG; HC03: 19.2; Pa02/Fi02: 236.7. Chest radiograph showed diffuse bilateral pulmonary infiltrates, predominantly in the right upper lobe and lingular segments. Given these findings and the patient's clinical presentation, computed tomography angiogram (CTA) of the chest was done which was negative for acute pulmonary embolism but showed patchy, nodular ground glass opacities along a bronchovascular distribution throughout the lungs (Figure 1(a)).

The patient was started on intravenous levofloxacin 750 mg daily and trimethoprim-sulfamethoxazole 400 mg every 6 hours since the suspicion for an opportunistic infectious pneumonia was high. She was also started on systemic corticosteroids, prednisone 80 mg daily. Bronchoscopy with bronchoalveolar lavage was performed, with bacterial, fungal,* Pneumocystis jiroveci*, and tuberculosis cultures being negative. The bronchoalveolar lavage revealed red blood cells: 100; white blood cells: 576; segmented cells: 22%; lymphocytes: 59%; and monocytes: 1%. Nasal swabs for influenza A and B, parainfluenza 1 and 3, adenovirus, and RSV were negative. A transbronchial left lower lobe lung biopsy was also performed. Pathology results from lung biopsy showed a single small fragment of alveolate pulmonary parenchyma showing no histopathologic abnormality. There was no evidence of inflammation, alveolar thickening or filling, or malignant infiltrates. There were no granulomas or vasculitis identified. Blood cultures were also negative. The patient's clinical condition improved rapidly. Given the high suspicion of brentuximab related acute lung injury, further treatments were discontinued. The patient was discharged on levofloxacin 750 mg daily, to complete a total of 14 days. She was also discharged on a prednisone taper, decreasing prednisone by 20 mg per week. Follow-up computed tomography of the chest four months later showed complete resolution of bilateral pulmonary opacities previously seen (Figure 1(b)) and ongoing remission of Hodgkin's lymphoma.

## 3. Discussion

We diagnosed brentuximab related acute lung injury based on the temporal association of pulmonary symptoms and the recent use of brentuximab and the documented resolution of the patient's symptoms after discontinuing brentuximab. This was further supported by excluding common infectious etiologies and excluding relapse of lymphoma and bleomycin related lung toxicity given the fact that the time elapsed since her last cycle of AVBD was greater than one year [3] and the patient had documented normal computed tomography (CT) of the thorax after conclusion of the treatment.

In the AETHERA trial, a patient was considered for brentuximab consolidation therapy if they met at least one of the following risk factors for progression after ASCT: primary refractory Hodgkin's lymphoma; relapsed Hodgkin's lymphoma with an initial remission duration of less than 12 months; or extranodal involvement at the start of pretransplantation salvage chemotherapy [2]. Our patient was placed on brentuximab therapy due to relapsed Hodgkin's lymphoma with an initial remission duration of 4 months.

As the risk of recurrent pulmonary toxicity was felt to be high, the patient was not rechallenged and further treatment with brentuximab was discontinued. Currently, there is very little clinical data reported in the literature in regard to the clinical presentation of patients suspected of having pulmonary toxicity due to being exposed to the antibody-drug conjugate. Given our patient's presentation, we recommend any patient presenting with nonspecific pulmonary symptoms who is actively receiving or previously received brentuximab be evaluated to rule out infectious etiologies. If infectious etiologies are negative stopping brentuximab may improve patient outcomes and prevent further lung toxicity.

Brentuximab is biochemically composed of the cytotoxic drug monomethyl auristatin E (MMAE), an antimitotic agent that inhibits tubulin polymerization, and an anti-CD30 monoclonal antibody [4]. Brentuximab binds and is internalized by cells that express the CD30 antigen; subsequently, MMAE causes cell cycle arrest and apoptosis [5]. The CD30 antigen is significantly present in the tumors of Hodgkin lymphoma and anaplastic large cell lymphoma; as such this antibody-drug conjugate is effective and specific for treating these disease entities [4]. Notably, brentuximab was shown in randomized clinical trials to significantly improve progression free survival in patients with Hodgkin's lymphoma who have risk factors for relapse or progression after ASCT [2]. As with other chemotherapeutic agents there are adverse effects with the use of brentuximab, the most common being peripheral sensory neuropathy (52%–56%), neutropenia (54–55%), fatigue (41%–49%), and nausea (38%–42%) [1, 2]. In clinical trials when brentuximab was combined with ABVD, up to 40% of patients were noted to have pulmonary toxicity, leading to a FDA “black box” warning [6]. Notably, only 5% of patients in recently published AETHERA trial, who were receiving consolidation brentuximab after stem cell transplant, developed pulmonary toxicities [2]. In randomized clinical trials, noninfectious pulmonary toxicity has presented variably as pneumonitis, interstitial lung disease, or acute respiratory distress syndrome, and it has caused fatal outcomes in some cases [6].

Other chemotherapeutic agents such as gemcitabine, carmustine, and bleomycin have been associated with lung toxicity. Bleomycin pulmonary toxicity is also known as bleomycin induced pneumonitis which is recognized mostly by the timeline of symptom onset. Typically symptoms begin one to six months after receiving bleomycin [3]. Reports have also shown that gemcitabine pulmonary toxicity incidence ranges from 0.7% to 13% in retrospective analysis [7]. Pulmonary lung toxicity has presented with diffuse alveolar damage, acute respiratory distress syndrome, interstitial pneumonitis, or noncardiogenic pulmonary edema requiring steroid therapy. Also similar to brentuximab, patients with bleomycin and gemcitabine-induced pulmonary toxicity typically present with nonspecific subacute clinical syndromes, for example, dyspnea, cough, fever, and fatigue; thus a high index of suspicion is needed when the diagnosis remains elusive after an extensive workup [8, 9]. In contrast, the diagnosis of carmustine lung toxicity is supported by a lung biopsy which typically shows interstitial fibrosis with edema and hyaline membrane formation [10]. Treatment for this particular patient was mostly supportive with the use of oxygen and nebulizers; interestingly, some reports have shown that steroids are the most effective treatment [8].

## 4. Conclusion

To the best our knowledge, this is the first case reported that provides the best detailed description of the clinical presentation, clinical characteristics, and a reasonable therapeutic approach to a patient with a serious adverse effect related to one of the newest chemotherapeutic agents in the field of hematology/oncology. We would like to inform the busy clinician about this rare but potentially serious and lethal adverse effect that can occur in patients being exposed to brentuximab for the current therapies in Hodgkin's lymphoma.

## Abbreviations

ASCT: Autologous stem cell transplant
CTA: Computed tomography angiogram
MMAE: Monomethyl auristatin E.
